# Assessing Visual Attention Using Eye Tracking Sensors in Intelligent Cognitive Therapies Based on Serious Games

**DOI:** 10.3390/s150511092

**Published:** 2015-05-12

**Authors:** Maite Frutos-Pascual, Begonya Garcia-Zapirain

**Affiliations:** DeustoTech Life [eVIDA] Faculty of Engineering University of Deusto, Avda de las Universidades 24, Bilbao 48015, Spain; E-Mail: mbgarciazapi@deusto.es

**Keywords:** eye tracker, attention, intelligent therapies, serious games, children

## Abstract

This study examines the use of eye tracking sensors as a means to identify children's behavior in attention-enhancement therapies. For this purpose, a set of data collected from 32 children with different attention skills is analyzed during their interaction with a set of puzzle games. The authors of this study hypothesize that participants with better performance may have quantifiably different eye-movement patterns from users with poorer results. The use of eye trackers outside the research community may help to extend their potential with available intelligent therapies, bringing state-of-the-art technologies to users. The use of gaze data constitutes a new information source in intelligent therapies that may help to build new approaches that are fully-customized to final users' needs. This may be achieved by implementing machine learning algorithms for classification. The initial study of the dataset has proven a 0.88 (±0.11) classification accuracy with a random forest classifier, using cross-validation and hierarchical tree-based feature selection. Further approaches need to be examined in order to establish more detailed attention behaviors and patterns among children with and without attention problems.

## 1. Introduction

In recent years, the usage of video game-related content in areas, such as education, therapies and training, has risen sharply. Several studies suggest that the future of pedagogy will inevitably be linked to the proposal of combined play and learning in order to promote creativity in future generations [1]. The boom in serious games brings together the potential available invideo games, devoting it fully to the enhancement of specific abilities, skills and aptitudes in children and adults.

Moreover, the design and development of new adaptive serious games whose content changes based on user interaction make therapies, training and education more customized. These techniques provide systems with an efficient way of learning based on the users themselves, providing them with customized and personal experiences, which may increase their potential effects [2].

One of the most widely-used forms of adaptive intervention consists of helping students to complete some educational activities when they have specific difficulties proceeding on their own [3].

The purpose of this study is to explore the use of eye tracking sensors to evaluate the behavior of children in attention-related cognitive therapies based on serious games to determine the utility of eye-related data as an input biofeedback signal for attention improvement therapies.

Eye movements are a natural information source for proactive systems that analyze user behavior, where the goal is to infer implicit relevance feedback from gaze [4]. Moreover, following the eye-mind hypothesis put forth by Carpenter in 1980, there is a close link between the direction of the human gaze and the focus of attention [5], provided that the visual environment in front of the eyes is pertinent to the task that we want to study [6]. Eye tracking sensors collect information about the location and duration of an eye fixation within a specific area on a computer monitor.

In this study, normal developing children aged between eight and 12 years and with different attention skills are asked to solve a set of puzzles while their gaze patterns and interaction are recorded using an eye-tracking sensor. The recorded eye information includes the location of gaze fixation on the computer screen, the duration of fixations and saccades (the path of the eye movements), along with interaction information regarding performance during the exercise. We hypothesize that participants with better performance in the proposed exercises would demonstrate patterns of eye-movements that are quantifiably different from individuals with a weaker performance. Identification of these differences would be especially advantageous for teachers and psychologists, as this study may provide new insight into the strategies for the improvement of attention skills. Moreover, the authors would like to study the relation between gaze patterns and the degree of expertise. This will be done by determining if there are any differences between the first approach to an exercise and the subsequent ones.

This article is outlined as follows: First, the use of eye tracking sensors in the field of serious games will be studied and placed in context. Subsequently, the Materials and Methods Section will be introduced, in which the authors discuss the form and function of the data collected from the eye tracking sensor. Next, a discussion of the collected data and the approaches to data analysis are examined. Finally, the manuscript concludes with a discussion of possibilities for further research into the uses of eye tracking sensor and data as a biofeedback input to intelligent therapies.

## 2. Literature Review

The observation of eye-movements is not a new area of research within psychology-related fields, having been studied in depth over the last few decades [7–9].

Research using eye tracking sensors affords a unique opportunity to test aspects of theories about multimedia learning concerning processing during learning [10]. Moreover, the use of this approach may help in understanding where players focus their attention during game play [11], as well as how they confront unfamiliar games and software [12].

However, it was not until recently that researchers began to analyze and introduce eye tracking sensors and techniques in serious games and computer games [13–15]. Games that can be controlled solely through eye movement would be accessible to persons with decreased mobility or control. Moreover, the use of eye tracking data can change the interaction with games, producing new input experiences based on visual attention [15].

Eye tracking devices have been used in the design of educational games, in terms of assessing usability based on user gaze behaviors when interacting with the game [16,17]. El-Nasr and Yan used eye tracker sensors to analyze attention patterns within an interactive 3D game environment, so as to improve game level design and graphics [18].

Kickmeier-Rust *et al.* focused on assessing the effectiveness and efficiency of serious games. For this purpose, they assessed these variables with gaze data and gaze paths, in order to obtain interaction strategies in specific game situations [19]. Sennersten and Lindley also evaluated the effectiveness of virtual environments in games through the analysis of visual attention using eye tracking data [20]. Johansen *et al.* discussed the efficiency of eye tracker sensors in assessing users' behavior during game play [21].

Józsa and Hamornik used recorded eye tracking data to evaluate learning curves in university students while using a seven hidden differences puzzle game. They used this data to assess similarities and differences in information acquisition strategies considering gender- and education-dependent characteristics [22]. Dorr *et al.* conducted a similar study concluding that expert and novice players use different eye movement strategies [23]. Muir *et al.* used eye-tracking data to capture user attention patterns and to present results on how those patterns were affected by existing user knowledge, attitude towards getting help and performance while using the educational game, Prime Club [3].

Radoslaw *et al.* used eye tracker sensors for assessing render quality in games. They argued that gaze-dependent rendering was especially important when immersed in serious games, where players in virtual environments played a primary role [24]. Smith and Graham and Hillaire *et al.* concluded that use of an eye tracker increases video game immersion, altering the game play experience [25,26].

Chang *et al.* developed the game WAYLAas a means to evaluate the potential to offer new interaction experiences based on eye tracking and visual attention. These authors took advantage of the popularity and arrival of more affordable eye tracker sensors [27].

Li and Zhang used eye-movement analysis to assess patients' mental engagement in a rehabilitation game. Therapists use this feedback to adjust rehab exercises to users' needs [28]. Continuing with the health-related field, Lin *et al.* developed an eye-tracking system for eye motion disability rehabilitation as a joystick-controlled game [29]. Vickers *et al.* developed a framework that integrated automatic modification of game tasks, interaction techniques and input devices according to a user ability profile [30].

Walber *et al.* presented EyeGrab, a game for image classification controlled by the players' gaze. The main purpose of this game was to collect eye tracking data to enrich image context information [31].

Other studies, such as those conducted by Nacke *et al.*, evaluated the use of eye tracker sensors as an alternative way of controlling interaction with games, obtaining favorable outcomes where this challenge results in positive affection and feelings of flow and immersion [32]. Ekman *et al.* goes one step further, discussing the limitations of using pupil-based interaction and providing suggestions for using pupil size as an input modality [33].

Table 1 shows the experimental conditions for the most relevant articles included in this section.

## 3. Materials and Methods

This section presents the methodology used in this study along with participants' characteristics and the selection procedure.

### 3.1. Participants

The process for assessing attention was performed with a group of typically developing children. This process relies on data recorded with an eye tracking sensor. Participants were aged between 8 and 12 years, with an average age of 10.0 (SD = 1.34). Thirty-two randomly-selected participants (13 girls and 19 boys) were selected from a group of 83 volunteers by their teachers. This sample size was considered adequate for the purpose of the outlined pilot study [34].

These children live in the Basque Country, Spain, have not been diagnosed with any attention-related disorder and speak Spanish as their mother tongue. All of the participants were recruited from the Colegio Vizcaya School.

Since they were mature minors, the approval of parents or guardians was requested prior to conducting the study. This approval consisted of an informed consent following receipt of a detailed description of the study, distributed via the school's regular newsletter.

### 3.2. Materials

**Figure 1:**
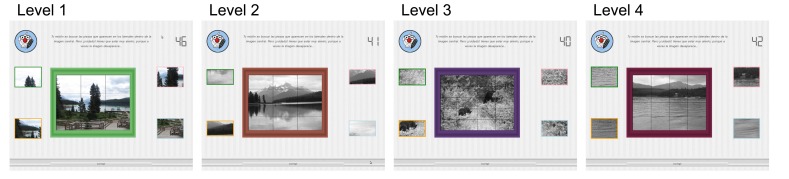
Different levels of the task.

All participants in the study completed the same assessment, which consisted of a puzzle exercise with four different levels of difficulty. Users have to connect each of the four slices presented in the exercise with its corresponding part in the main image. As Figure 1 displays, all of the participants were presented with the same image for each level, and all of the elements in the user interface appeared in the same part of the screen at each level.

The main image and the slices appeared in the middle of the screen, occupying the whole display from left to right. The question stem appeared in the upper middle part of the screen. The button to advance to the next level appeared at the lower middle part of each screen. The consistent layout of the screen was intended to minimize wide eye movements.

Different levels' settings are outlined in Table 2. All of the users had a maximum pre-set time of 50 seconds to complete each of the levels. However, if they finished the level before the time ended, they could go on to the next exercise. Depending on the level, the displayed image was labeled as easy, medium or hard. Only Level 1 is displayed in color. Hard images have very similar slices and are more complicated to complete. Table 2 displays the different levels’ settings.

### 3.3. Devices and Technologies

All of the data for this study were collected on the same device, which was located at the children’s school outside the laboratory environment. These conditions were considered appropriate due to the nature of the system.

The set of puzzles was developed in Python [35]. The results obtained and the necessary parameters were stored in a SQLitedatabase [36]. The user interface and user interaction were developed using PyQT4 [37]. Fixation heat maps were produced based on the implementation developed by jjguy [38]. The classification process was implemented using the Scikit-learn library for machine learning in Python [39].

**Figure 2:**
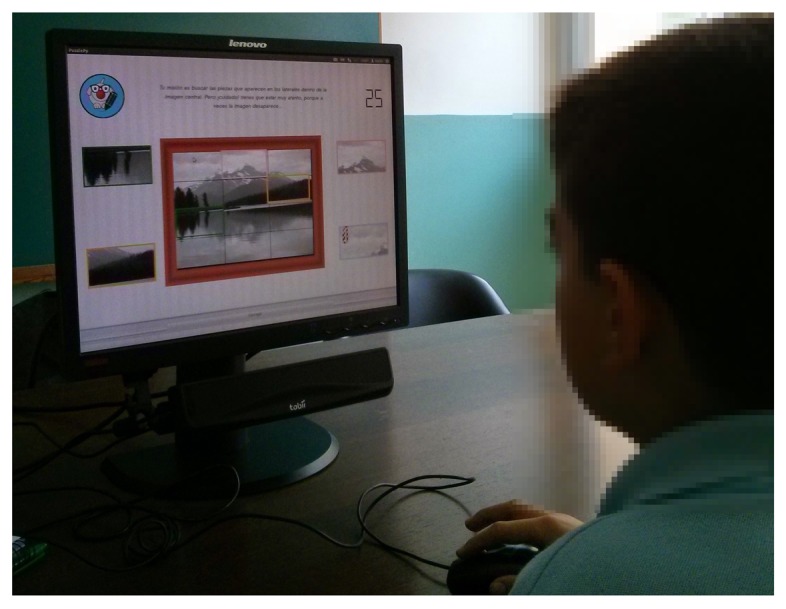
Participant using the system while his gaze is being recorded.

The puzzles were displayed on a 19-inch Lenovo monitor interface with an Acer Aspire Timeline X laptop running on Ubuntu 12.04. All of the text in the different exercises was displayed as black text against a light-grey background following normal grammatical conventions in Spanish. Images were inserted as JPEG digital pictures scaled from their original versions. Response selection and any changes were stored by monitoring the user interaction and recording eye movements with a Tobii X1 Light eye tracker sensor. Figure 2 shows the study setting while one of the participants was interacting with the system.

The eye tracker is a non-invasive sensor with remote function. Participants were not required to remove their glasses or contact lenses during the tests. Accuracy under ideal conditions is 0.5 deg of the visual angle, while the sampling rate in this study was typically 28–32 Hz. As Figure 2 displays, the Tobii X1 light sensor was located beneath the computer monitor with the headrest fastened to the front edge of the desk, monitoring the participant's head. The laptop was located behind the monitor, without interfering with the participants' field of vision.

A typical experimental trial including calibration lasted less than 20 min for each participant.

### 3.4. Experimental Procedure

Prior to this study, participants' teachers responded autonomously to the EDAH scale for the evaluation of ADHD in the questionnaire on children between 6 and 12 years old [40]. Farré and Narbona designed this scale based on their experience with the adapted Conners questionnaire [41]. The EDAH measures the main characteristics of ADHD and the behavioral problems that may coexist with attentional deficit. This questionnaire was used to ensure that participants did not exhibit any ADHD-related behavior.

After completing the exercises, participants themselves were asked to fill in a usability questionnaire. The usability of the system was evaluated by a user satisfaction test based on the System Usability Scale [42]. This questionnaire consists of 10 items, which were evaluated by using a Likert scale ranging from 1, strongly agree, to 5, strongly disagree. Through feedback from this questionnaire, researchers will be able to continue to adapt the system to users’ final needs.

Before completing the usability questionnaire, participants were seated in front of the eye tracking sensor to permit data collection. Users were seated opposite the center of the monitor, after adjusting the seating position to their height. Once they were aligned with the screen, the calibration process started, which took between 2 and 5 min per child. This calibration entails a visual target that moves around the screen. Participants were asked to follow this target with their gaze for a period of time. The target consists of a calibration grid with 5 positions, one on each corner of the screen and the last one right in the screen’s center. The target consists of different calibration bullet points that appeared one after the other in the same order for all participants, starting from the top left corner.

Prior to the start of the exercises, participants were told in which kind of tasks they were taking part. They were also introduced to the eye tracking technology, and the sensor functionality was explained.

Participants used the system and filled in the questionnaire in a controlled environment, with a researcher observing and keeping track of all of the behavioral aspects of the study, but not interfering in the experimental setting.

### 3.5. Data Analysis, Processing and Classification

**Figure 3:**
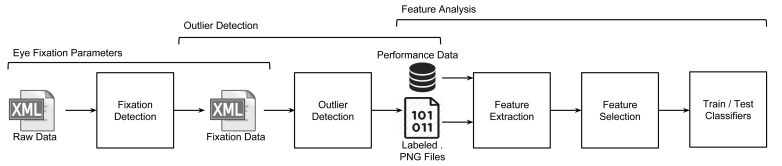
Raw data processing.

Recorded gaze data during the exercises has been processed, analyzed and used in order to identify the set of features that may help to build a classifier, as shown in Figure 3.

This section will explain in detail the different steps involved in the data analysis and feature identification process, so as to contribute to the core of intelligent therapies based on visual attention and user interaction.

#### 3.5.1. Eye Fixation Parameters

The analysis of fixations and saccadic movements during the performance of certain tasks is related to attention in various ways. Several studies support this hypothesis [43-45], concluding that oculomotor mechanisms rely on attention for some aspects of eye movement control [46].

During the performance of the study, raw gaze data were recorded with the eye tracking sensor. These raw gaze data were stored as .xml files in the system, with information related to the level of the exercise that was currently running.

Listing 1 shows the stored gaze data for each participant and exercise. These data consist of the (x, y) coordinates recorded by the eye tracking sensor, the timestamp in which they were perceived, the pupil size for each eye and the exercise; the level and the mode the coordinates belong to were also stored for matching the raw gaze data with other interaction recordings.

These raw data were used for analysis and processing so as to obtain meaningful information about eye fixation locations, fixation durations, saccades and saccadic durations. Fixations are the period of time when the eyes remain fairly still and new information is acquired from the visual array [9], while saccades are the eye movements themselves. During saccades, no information is retrieved by the brain, since vision is suppressed under most normal circumstances [47].

Listing 1: Raw gaze data example
1<user date = "2014–05–22" id = " 12" sessionid = "899" time = "09:20:15" >2 <exercise id = "puzzle" level = "Level5" mode="performance">3  <eyedata>4   <timestamp time = " 1167610729217997 ">5    <left_eye pupil_diam = "3.0435333252" validity = "0" x=" 134.831732304" y=" 64.8299084174"/>6    <right_eye pupil_diam = "2.89215087891" validity = "0" x=" 69.9537996816" y=" 59.426052033"/>7   </timestamp>8   …9  </ eyedata>10 </ exercise>11</user>

**Figure 4:**
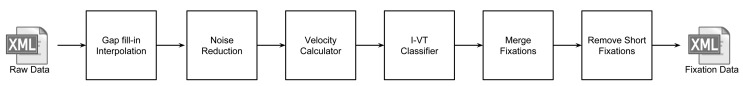
Raw data processing [48].

In order to detect the saccades and fixations, some processing techniques need to be applied to the raw data file. These steps are based on the Tobii I-VTfixation filter algorithm [48], have all been implemented in the Python programming language and are outlined in Figure 4.

As Figure 4 shows, the first step in the processing algorithm is to apply the gap fill-in interpolation function. This step consists of filling in data where data are missing due to tracking problems that are not related to participants’ behavior (such as blinks or when the user looks away from the screen). In order to distinguish between tracking problems and users’ behavior, a max gap length is set, which limits the maximum length of the gap to be filled in. Following Tobii’s white paper for the I-VT fixation filter and the value used by Komogortsev, this value was set at 75 ms [48,49].

After the gaps are filled in, the noise reduction function is applied. This function is based on a low-pass filter, which aims to smooth out the noise. The third step is the velocity calculator, which relates each sample with its velocity, in terms of visual angle (degrees per second). In order to reduce the impact of noise, the velocity for each sample is calculated as the average velocity of a period of time, taking as the central data input the current sample. This is done using a window length of 20 ms, which, according to the literature, has been found to handle a reasonable level of noise without distorting the signal [48].

The I-VT classifier applied to the signal is based on the one described by Komogortsev *et al.* [49] and outlined in the Tobii white paper [48]. The classifier determines which samples belong to a saccade, fixation or gap, based on a velocity threshold and the angle velocities calculated in the previous step. It also groups together consecutive samples using the same classification. The velocity threshold is set to 30 deg/s [48,50].

The merge fixations function aims to merge adjacent fixations that have been split up. This is done taking into account two different thresholds, the max-time between fixations, which is set to 75 ms [48], that is lower than the normal blink duration [49,51,52], and the max-angle between fixations, which is set at 0.5 deg [48,49,53–55]

Once all of the fixations have been identified, the shorter ones are removed. For the purposes of this analysis, 100 ms was set as the lower limit for fixation duration. This value was chosen based on the work of McConkie *et al.*, who concluded that 60 ms must pass before current visual information becomes available to the visual cortex for processing [56]. R. Tai *et al.* arrived at the lower limit of 100 ms by adding 30 ms, which is the time that elapses, at the end of a fixation, between when a command to move the eyes is sent and the onset of that saccade is reported. They allowed also 10 ms for the processing of any currently-observed stimuli, arriving at the 100-ms threshold [57].

After all of the processing functions have been applied to the current data, a new gaze data file is created with all of the fixations for the current exercise and participant. As shown in Listing 2, fixation data have a similar structure to raw data.

Listing 2: Fixation data example
1<user date="2014–05–22" id="12" sessionid="897" time="09 :15:27 ">2 <exercise id=" puzzle " level=" Level1 " mode=" performance ">3  <fixationData>4   <f ixation >5    <time duration =" 212.356933594 " end_time=" 1.1676104137 e+12" start_time =" 1.16761041349 e+12">6     < position x=" 54.2251062717 " y=" 130.508713537 " />7    </ time>8   </ fixation>9   …10  </ fixationData>11 </ exercise >12</ user>

The stored fixation data save all of the fixations recorded during the exercise, along with the current activity information, user data and the duration, start time, end time and position of each fixation.

#### 3.5.2. Outlier Detection Process

**Figure 5:**
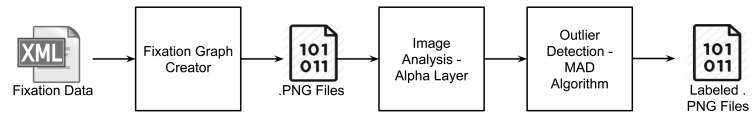
Outlier detection process.

Once the processing stage is over, the fixation data are used to determine the outliers among the recorded data. This process is outlined in Figure 5.

As Figure 5 shows, a fixation heat map is created for each file. The fixation count heat map shows the accumulated number of fixations for each puzzle level and for each participant. Each fixation made adds a value to the color map at the location of the fixation [58].

The alpha layers of the images stored are then analyzed as a measure to identify the location and amount of fixations and saccades. All of the images are the same size and dimensions.

The alpha information per image is stored in order to be processed by the median absolute deviation (MAD) algorithm for outlier detection implemented in Python.

The median deviation is a measure of scale based on the median of the absolute deviations from the median of the distribution [59]. The formula is shown in Equation (1).

$$\text{MAD}={\text{median}}_{i}\left(\left|X_{i}-{\text{median}}_{j}\left(X_{j}\right)\right|\right)$$

Moreover, the heat maps were analyzed taking into account users’ overall performance during the entire study, so as to have another feature to determine outlier detection.

#### 3.5.3. Classification

This section outlines the first steps taken in the classification process. The aim of this part is to assess the feasibility of using a set of combined features to evaluate user performance. These features are related to user interaction, timing and visual attention, as well as image-related data obtained directly from the heat maps.

This part explains the theoretical insights taken in this process. Please refer to the same section in the Results part for the mathematical outcomes of this process.

##### 3.5.3.1. Feature Identification

Feature selection is a determining factor when classifying patterns. Features need to be insensitive to noise and separated from each other. Their main purpose is to objectively describe certain aspects, in this case of the attention and performance process in intelligent therapies aimed at children.

A collection of 34 features was selected based on image characteristics and user performance related to the current exercise. Features were selected based on the recorded data. The authors, in conjunction with the multidisciplinary team taking part in this project, took into consideration performance variables, as well as gaze pattern recordings. The subset of selected features for analysis from the pilot phase is outlined in Table 3.

Heat maps were divided into 9 quadrants in order to obtain detailed data about the location and density of fixations per participant and level.

The selected features were chosen for further analysis and consideration, so as to determine if they are suitable for use in an automatic classifier, capable of discerning the users' performance based on their interaction and gaze patterns.

##### 3.5.3.2. Feature Selection

Feature selection creates a subset of features, improving their predictive performance and constructing patterns more efficiently. This helps to avoid multidimensionality, which may otherwise have an adverse effect on the decision making process [60].

Several techniques were used in this process. In order to assess the success rate of the classifier while obtaining the most accurate set of features, a set of different ensemble classifiers was used and compared with a traditional decision tree classifier.

- Sequential search: This process works by selecting the best features based on univariate statistical tests [39]. Inside this topic, the select *k*-best feature selection algorithm was applied. This process removes all but the *k* highest scoring features.
- L1-based feature selection: This was applied to assess the feasibility of discarding the zero coefficients. This is a means of reducing the dimensionality of data [39].
- Hierarchical feature selection: In these feature selection processes, the set of features is divided into smaller subsets until only one remains in each node [61]. Tree-based estimators were applied to compute feature importance, so as to discard the irrelevant ones [39].

##### 3.5.3.3. Classifier Performance Analysis

Ensemble learning algorithms works by running a base learning algorithm multiple times, voting out the resulting hypotheses [62]. Ensemble learning has received an increasing interest recently, since it is more accurate and robust to noise than single classifiers [63,64].

This article compares the performance capabilities of 3 different ensemble algorithms when they are applied to the real dataset recorded in this study. The aim of this experiment is to assess the feasibility of building a classifier able to determine user performance using an adequate set of features of a different nature recorded during the therapy.

All of the classifiers were evaluated using cross-validation. The studied classifiers were:

- Random forest: This classifier is defined as a combination of tree predictors. Each tree depends on the values of a random vector sampled independently and with the same distribution for all trees [65]. Using the random selection of features yields error rates that compare favorably to AdaBoost [66], but are more robust with noise handling [65].
- Extremely randomized trees: A tree-based ensemble method for supervised classification and regression. It is a strongly randomized attribute selection method. This algorithm is accurate and computationally efficient [67].
- AdaBoost: This algorithm is an iterative procedure that tries to approximate the Bayes classifier by combining several weaker classifiers. A score is assigned to each classifier, and the final classifier is defined as the linear combination of the classifiers from each stage [68].

Moreover, a regular decision tree classifier was applied in order to assess the potential and improvement in accuracy, if any, of the previously mentioned tree-based ensemble methods.

## 4. Results

The recordings for the results explained in this section were taken during the month of May, 2014, at the Colegio Vizcaya school in Biscay, Spain.

### 4.1. Analysis of User Performance: Outcome Scores and Response Times

Although the present study is focused on the use of gaze data to analyze performance in attention-related cognitive therapies, we feel that it is also important to address commonly-used measurements to categorize user performance in this type of exercise: outcome scores and response times. These measures might be quite general in some cases where they show only a vague impression of the user's performance.

Participants' responses were recorded through the system implemented in Python. Their overall number of correct responses, as well as their number of correct responses per level are shown in Table 4.

The overall mean of correct responses is 11.937 (SD = 2.20) out of a possible score of 16. When the results are examined by levels, there are some differences in performance between the first two levels, which participants considered much easier, and the last two, which they found more difficult.

Users had a maximum of 50 seconds to complete each exercise. However, they were able to finish the level before time ran out. Considering the response times, *i.e.*, total time spent on test questions, the data show that the majority of participants took most of the entire time available at all of the levels.

Levels can be segmented into two groups, according to difficulty. The first two are considered the easiest ones, while the last two are trickier. There is a tendency between the two groups; users tend to perform slower with Levels 1 and 3 than with Levels 2 and 4. This may be because they tend to be more careful with novelty exercises or when the difficulty suddenly changes.

**Figure 6:**
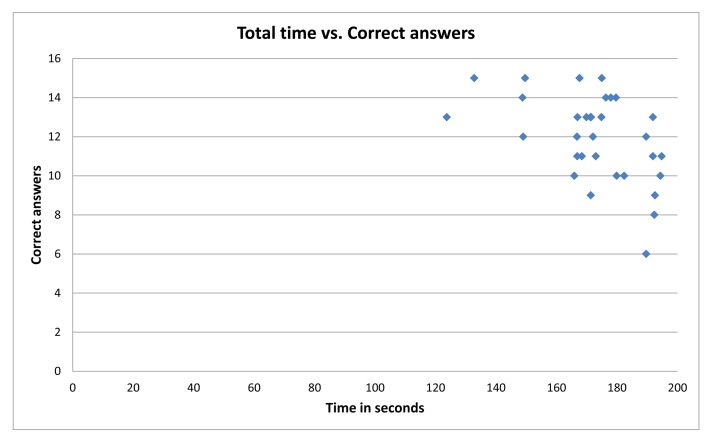
Time vs. correct answers: user performance.

Figure 6 shows the overall performance of participants, regarding total time *versus* correct answers. As is displayed in Figure 6, users tend to respond correctly to more than half of the possible answers, while using 75% or more than the available time. When analyzing the group with the weakest performance, with a number of total correct answers below 10, it is clear that 75% of the participants in this group have a higher performance time.

Further analysis of user performance will be outlined in the following sections. The correct items' mean (11.93 out of 16) and standard deviation (SD = 2.20) values were used for obtaining the threshold for the weakest performers. This results in the value 9.73; since the study needs an entire threshold, this value was rounded up to 10. Participants with scores lower than this threshold were classified as the weakest performers. A total of four participants matched this criteria, so they were paired with the four best performers to obtain two balanced groups for further analysis. In order to address the research question stated in the Introduction, the four best performers (users with IDs 20, 25, 28 and 43) and the weakest four (users with Is 15, 16, 29 and 36) will be analyzed.

### 4.2. Fixation Heat Maps

Fixations were analyzed for each of the participants. Fixations were defined as a gaze longer than 100 ms. In order to address the research question stated in the Introduction, the most accurate and the weakest performers were selected for further analysis.

**Figure 7:**
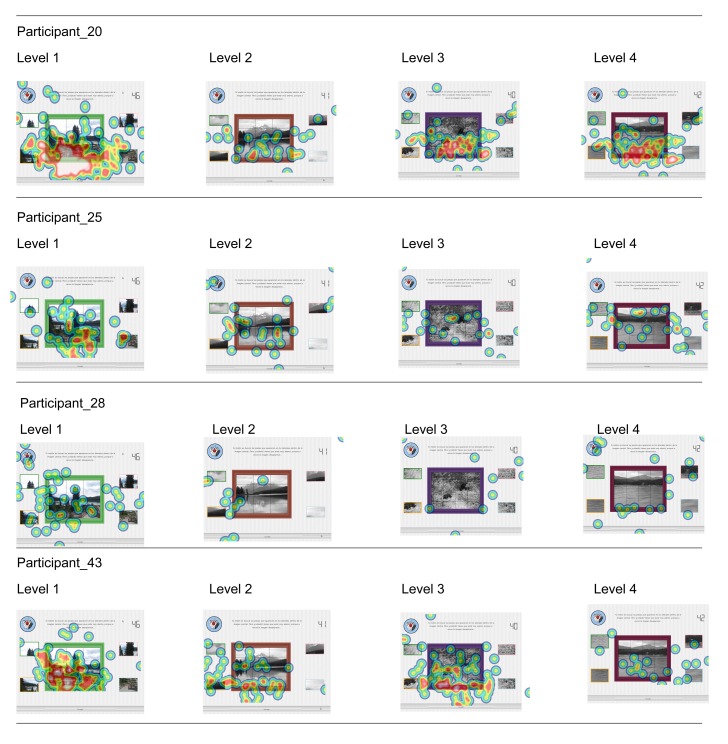
Participants with the best performance results.

**Figure 8:**
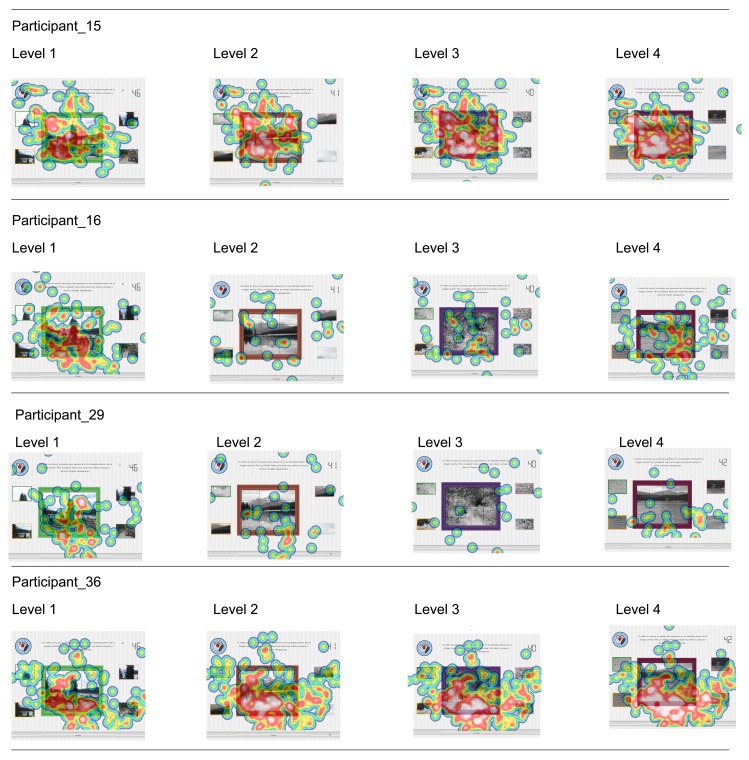
Participants with the worst performance results.

Fixations were displayed as heat maps, which were created based on the entire time participants took for each level. Red spots indicate higher levels of fixation, with yellow and green indicating decreasing amounts of fixations. Areas without color were not fixated upon. The most accurate performers are displayed in Figure 7, while the four with the weakest performance are displayed in Figure 8.

When comparing the heat maps of both groups, there are some differences between the number, density and clustering of fixations. In Figure 7, where the total score results of the participants are 15 correct answers out of 16 possible ones for every case, the number of fixations is lower than for the participants with a weaker performance. Not only is it lower among participants, it also seems to decrease when analyzing the intra-level gaze behavior for each of them.

It is important to bear in mind that an overall lower number of total fixations suggests less time spent viewing specific areas of the assessment item.

Regarding Figure 8, where the total score for these participants ranges between six and nine correct answers out of 16 possible ones, the fixation density is higher for all the cases, except for the participant with ID 29.

This hypothesis agrees with R. Tai *et al.*, who found an inverse relation between the fixation and saccade amount and the degree of expertise of the participants [57].

### 4.3. Quantitative Analysis

This section includes a quantitative analysis of the data regarding various features, such as the number of fixations per level, their average duration and the gender and age of the selected subgroups of participants, in order to analyze the feasibility of establishing some behavioral patterns.

**Figure 9:**
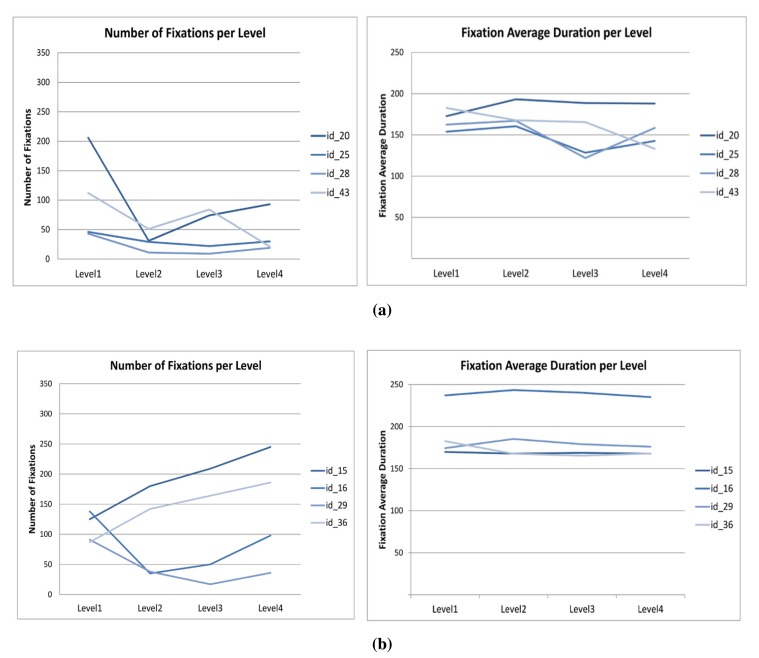
Fixation data: best and weakest performers. (**a**) No. of fixations vs. fixation avg. duration, best performers; (**b**) No. of fixations vs. fixation avg. duration, weaker performers.

Table 5 displays the four participants with the best results. When processing the number of fixations, we observe that they decrease in number with the progression of the levels for all of the users, as displayed in Figure 9a. Since the exercise has the same visual layout for every level, this may be related to their having achieved a certain degree of expertise with each new level.

Table 6 displays the four participants with the weakest performance results. When processing the number of fixations, we find no specific relationship among them either, as displayed in Figure 9b, which may be related to the lack of appropriate techniques for solving the puzzle task.

Further analysis of the results was made on the best vs. weakest performers' data. Due to the number of users that were used for the further analysis of the results, a Mann–Whitney non-parametric test was applied. The results of the test are outlined in Table 7.

Table 7 shows that there are some significant differences (*p* ≤ 0.05) in performance between groups. These differences appeared in the number of fixations in Levels 2 and 4 and globally. Moreover, there are other significant differences for fixation average time (global), time (Level 4) and the number of correct answers (Levels 3 and 4). However, these results may not be enough to conclude that there are consistent differences regarding the level of expertise of the participants.

R. Tai *et al.* [57] and Chi *et al.* [69] hypothesized that fixation duration data did not produce clear and consistent differences regarding the level of expertise of the participants, which agrees with the results obtained in this section.

### 4.4. Classification

This section outlines the first steps taken in the classification process. The aim of this part is to assess the feasibility of using a set of combined features to evaluate user performance. These features are related to user interaction, timing and visual attention, as well as image-related data obtained directly from the heat maps.

This part explains the mathematical outcomes of this process. Please refer to Section 3.5.3 for the theoretical insights.

In order to further assess the number of optimal features for the classification part, a recursive feature elimination process with cross-validation was applied. Table 8 displays the existing relation between the number of features and the classifier's accuracy. The number of features depends on the feature selection algorithm applied. These algorithms were outlined in Section 3.5.3.2.

The accuracy results displayed in Table 8 were obtained by applying a cross-validation process of 100 iterations to all of the available data. These user data were divided as follows: 60% of the data for training and 40% for testing the classifier inside the cross-validation process.

With this setting, feature selection seems to be beneficial for building any type of analyzed classifier. However, when employing all of the available features, the accuracy rate falls below 0.80 for decision trees and AdaBoost classifiers. The select K-best features algorithm improves the classifiers' accuracy, especially when using 22 features. The L1-based algorithm displays good accuracy results for all of the ensemble methods and falls below 0.80 for the decision tree classifier. The tree-based hierarchical algorithm employed gives good results in accuracy with a limited number of features that range between 10 and 14, depending on the classifier employed.

The authors compared the accuracy performance of the selected ensemble classifiers with the overall performance of the a decision tree classifier. Since the data did not follow a normal distribution, a Mann–Whitney analysis was used. The results of comparing the performance of every ensemble classifier (with all features) with the decision tree classifier (with all features) is displayed in Table 9.

As is displayed in Table 9, the use of ensemble classifier methods significantly improves the overall performance of the classifier, regardless of the number of features employed. In the case of using all of the available features, the best classifier for the recorded data is the random forest.

Analyzing the difference in intra-classifier performance, Table 10 displays the Mann–Whitney analysis of the different feature-selection algorithms, comparing their performance with the accuracy obtained with the all features approach.

Table 10 illustrates that for almost all of the analyzed settings in this article, the use of a smaller set of features significantly improves the overall accuracy of all of the ensemble classifiers and the decision tree.

After carrying out all of the detailed experimental tests based on the recorded data, it can be concluded that accurate classification of different user performance according to their interaction and visual attention is possible.

## 5. Discussion and Conclusions

In the Conclusion, we intend to give an answer to the research questions outlined in the Introduction, as well as put forth new thoughts and trends about the present and future of assessing visual attention using eye tracker sensors in serious games.

According to the literature, there are several theories that link eye-movements with attentional processes [5,6], linking eye movements with cognitive processes, such as reading, visual search and scene perception. However, regarding intelligent therapies, eye movements do not always tell the whole story about the attentional process [70]. These resources should be complemented with other interaction records, as well as with relevant data about the participant. The higher the system information, the more accurate its customization to users' final needs.

In the Introduction, we hypothesized that participants with better performance may demonstrate patterns of eye-movements quantifiably different from individuals with weaker performance. Although some differences were found during the exercises, it is necessary to extend the study or to replicate it, in order to make stronger assumptions.

A comparison of the fixation duration data did not produce clear and consistent differences corresponding to the level of performance. These results corresponded with those related to the expertise level found by R. Tai *et al.* [57] and Chi *et al.* [69].

Regarding Figure 8, which shows the fixation heat maps for the weaker performers, fixation density is higher for all of the cases, except for the participant with ID 29. Moreover, the fixation density in Figure 7 decreases with the performance of new levels. These findings agree with R. Tai *et al.*, who found an inverse relationship between the fixation and saccade amount and the participants' degree of expertise [57].

When analyzing performance data, there are some differences between the two groups for which the puzzle levels are classified into according to difficulty. Table 4 shows the performance results. When changing the exercise type or level of challenge, users tend to spend more time and perform the exercise with taking more time to think. When the tasks are repeated, the ability level increases and the time to complete them drops. This may be related to the acquisition of specific problem-solving skills, which become more accurate with repetition. Further studies need to be carried out about the users' ability and performance capabilities in repetitive tasks.

Intelligent therapies that dynamically adapt themselves to users' needs and performance based on their interaction with the system have been proven to be efficient in terms of improvement comparisons [71]. A good set of collected data may provide improved means for obtaining adapted and efficient intelligent cognitive data. Researchers should be very careful with the selected and recorded features. Several different approaches need to be followed in order to obtain the most accurate set of performance data.

Moreover, a deeper analysis of timing per exercise may also prove to be interesting for study. As a future approach, the reading instructions stage will be separated from the performance of the exercise, so that we can obtain explicit performance timing, with and without the reading stage. This could give further information about whether there are any differences between the first performance of an exercise and the subsequent ones. This new approach may also help in further assessment of attention in the performance and instruction reading stages.

Reviewing the literature, there are several studies published linking the size of the pupils with cognitive processes [72–74]. Although, this response in the pupils is slow [75]. Current eye trackers measure pupil size and give it as another parameter, so it is easy to analyze this feature during the performance of tasks. This parameter was not analyzed in this study, and it may be an interesting additional feature in future research about this topic.

In recent years, the popularity of eye trackers has increased, and there are some open-source projects offering tools for gaze data analysis [76–80], while some manufacturers offer low-cost devices, such as the EyeTribe [81]. There are also several DIY approaches for building custom eye trackers [82–84]. The accuracy of these systems may sometimes be slightly inferior to high-end eye trackers, but they may be a viable solution for use outside the laboratory setting [85]. The use of eye trackers outside the research community may help to extend its potential with available intelligent therapies, bringing state-of-the-art technologies to users.

This study may expand in future directions, such as the design and development of the system, so that the tool includes new skills that continue along the lines of the current tool, for work on new capabilities, such as working-memory or processing speed.

Moreover, future lines should include the design and development of a robust classifier, with the selected features outlined in Section 3.5.3.1. The initial study of the classifier capabilities of ensemble methods with the available user data has produced positive results, especially when implementing a feature selection algorithm beforehand (see Section 4.4 for further information about the ensemble classifiers performance). Other classifiers need to be studied and tested, in order to consider others that may be more accurate, alone or in combination with others. This approach will help to create an autonomous system able to discern user implication based on visual attention and performance records.

Finally, some directions for the future are to replicate this study:

- with a greater number of users;
- with users with and without attention-related problems;
- developing a bilingual or trilingual tool that allows the study to be replicated in other areas in Spain and abroad where reported diagnosis of attention-related problems are significantly different from the Basque Country, Spain.

The use of gaze data constitutes a new information source in intelligent therapies that may help to build new approaches that are completely customized to final users' needs. Further studies need to be carried out in order to establish more detailed attention behaviors and patterns among children with and without attention problems. The replication of this study, along with the extension of the current system with new exercises, may help to build personalized performance profiles per user. These profiles may help in creating new customized therapies, while providing a new degree of information to the children themselves, therapists, psychologists, teachers and family.

## Figures and Tables

**Table 1:** Literature review: Experimental conditions.

**Authors**	**Citation**	**Year**	**Country**	**Game**	**Device**	**Display**	**Participants**			

							**Total**	**Female**	**Male**	**Age (SD)**
Kiili et al.	[17]	2014	FI	Animal Class	Tobii T60	17"	8	2	6	7–13 years
Chang et al.	[27]	2013	PT	Wayla	Tobii REX	–	–	–	–	–
Muir et al.	[3]	2012	US	Prime Club	Tobii T120	17"	12	6	6	10–12 years
Walber et al.	[31]	2012	DE	EyeGrab	–	–	24	7	17	15–32 years
Kickmeier et al.	[19]	2011	AT	80 Days	Tobii 1750	–	9	4	5	13 (1.61)
Józsa and Hammornik	[22]	2011	HU	7 Hidden Differences	Tobii T120	17"	43	14	29	19–26 years
Pretorious et al.	[12]	2010	ZA	Timez Attack	Tobii 1750	17" (1280 × 1024)	8	4	4	9–12 years over 40
Sennersten and Lindley	[20]	2010	SE	FPS computer game	Tobii 1750	–	–	–	–	–
Nacke et al.	[32]	2010	CA	Half-Life 2	Tobii T120	–	30	2	28	18.67 (4.26)
Hillaire et al.	[26]	2008	FR	Quake III	ASL6000	Cylindrical Screen (1280 × 1025)	8	0	8	25.8 (4.3)
Dorr et al.	[23]	2007	DE	Breakout Game	SensoMotoric IViewX Hi-Speed	20"	9	–	–	–
El Nasr et al.	[18]	2006	US	Game Soul Caliber	ISCAN ETL-500 (head-mounted)	–	6	–	–	20–30 years
Smith and Graham	[25]	2006	CA	Custom build scene	RED250	22" (1680 × 1050)	21	1	20	21–24 years

**Table 2:** Different levels’ settings.

	**Time (s)**	**Grid Size**	**No. of Slices**	**Display**		**Image Level**		
	
				**Color**	**Greyscale**	**Easy**	**Medium**	**Hard**
Level 1	50	3×3	9	X		X		
Level 2	50	3×4	12		X	X		
Level 3	50	4×4	16		X		X	
Level 4	50	5×4	20		X			X

**Table 3:** Selected features for analysis.

**Feature**	**Data Type**		**Feature**	**Data Type**		**Feature**	**Data Type**	**Feature**	**Data Type**
Classification	outlier/normal		Fixation avgduration	ms		Fixation No. in A1–C3 (9 features)	Integer	Time per level (4 features)	seconds
s		Fixation No. in A1–C3 (9 features)	Integer		Time per level (4 features)	seconds			
Global alpha	percentage		Fixation max duration	ms		Fixation avg duration in A1–C3 (9 features)	ms	Total correct answers	integer
s		Total correct answers	integer						
s		Fixation avg duration in A1–C3 (9 features)	ms		Total correct answers	integer			
s		Total correct answers	integer						
Fixation total No.	Integer		Fixation min duration	ms		Total time in exercise	seconds	Correct answers per level (4 features)	integer

**Table 4:** Participants outcome scores and response times for each level.

**Participant ID (*n* = 32)**	**Level 1**		**Level 2**		**Level 3**			**Level 4**		**Total (16 max.)**	

	**Time (s)**	**Items Correct**	**Time (s)**	**Items Correct**	**Time (s)**	**Items Correct**	**Time (s)**	**Items Correct**	**Time (s)**	**Items Correct**
12	40.58	4	34.62	4	47.94	2	48.13	3	171.28	13
15	36.79	4	36.83	2	48.66	0	49.03	3	171.33	9
13	34.76	4	48.28	4	48.29	3	48.33	3	179.67	14
14	40.87	4	30.72	4	48.06	2	47.29	3	166.95	13
16	44.99	4	48.77	1	48.35	0	47.53	1	189.66	6
17	47.48	4	48.08	3	48.05	4	48.26	2	191.89	13
18	48.81	3	47.71	3	47.82	2	38.03	2	182.39	10
19	42.01	4	33.93	4	47.95	2	24.86	4	148.77	14
20	43.62	4	40.49	4	47.99	3	42.85	4	174.96	15
21	29.76	3	24.35	4	48.12	2	21.47	4	123.72	13
22	31.21	4	33.36	4	43.60	2	40.81	2	148.98	12
23	48.71	3	33.31	4	48.09	4	46.19	3	176.32	14
24	39.25	4	41.33	4	48.24	1	39.53	2	168.37	11
25	25.63	4	29.38	4	48.03	4	29.73	3	132.79	15
26	30.11	4	48.50	3	48.28	1	39.94	3	166.84	11
27	48.90	3	44.77	4	48.07	2	47.92	3	189.67	12
28	43.09	4	41.57	4	47.94	3	35.00	4	167.61	15
29	48.53	4	48.12	3	47.82	0	48.07	2	192.56	9
30	48.65	4	37.18	0	41.56	4	39.36	4	166.77	12
31	39.92	4	33.26	3	47.76	1	44.94	2	165.91	10
32	48.72	4	46.81	3	48.09	2	48.25	2	191.89	11
33	42.77	4	46.85	4	47.90	2	37.31	3	174.85	13
34	46.62	4	31.48	4	47.93	3	43.86	2	169.92	13
35	48.60	3	48.95	4	48.42	2	48.79	2	194.78	11
36	48.69	3	46.78	3	48.29	1	48.57	1	192.34	8
37	49.08	4	48.84	3	48.52	1	47.88	2	194.34	10
38	35.96	4	46.81	4	46.56	3	48.62	3	177.97	14
39	46.30	4	39.33	3	47.39	1	46.84	2	179.88	10
40	40.34	4	36.38	3	48.07	3	48.20	1	172.99	11
41	38.17	4	45.31	3	48.08	2	39.87	4	171.45	13
42	48.50	4	27.59	4	47.95	2	48.02	2	172.07	12
43	48.03	3	23.81	4	38.89	4	38.89	4	149.63	15

Average (*SD*)	42.36 (6.70)	3.75 (0.42)	39.80 (7.97)	3.37 (0.94)	47.40 (2.09)	2.12 (1.18)	42.89 (7.16)	2.65 (0.94)	172.45 (17.18)	11.93 (2.20)

**Table 5:** Participants gender, age, number of fixations and average duration of these per level: best performers.

**Participant ID**	**Age**	**Gender**	**Level 1**			**Level 2**			**Level 3**			**Level 4**			**Total**	

			**No. Fix.**	**Fix. Avg Duration (ms)**	**SD (ms)**	**No. Fix.**	**Fix. Avg Duration (ms)**	**SD (ms)**	**No. Fix.**	**Fix. Avg Duration (ms)**	**SD (ms)**	**No. Fix.**	**Fix. Avg Duration (ms)**	**SD (ms)**	**Time (s)**	**Correct Ans**
20	9	Female	206	172.72	67.03	31	193.10	74.89	74	188.59	68.65	93	188.08	67.93	174.96	15
25	12	Male	46	153.85	58.53	29	160.49	70.62	22	128.40	37.64	30	142.66	72.09	132.79	15
28	11	Male	43	162.42	59.04	11	167.07	57.24	9	121.98	9.82	19	158.42	55.27	167.61	15
43	11	Female	112	182.61	50.90	51	167.70	90.66	84	165.43	78.35	21	133.15	39.96	149.63	15

**Table 6:** Participants gender, age, number of fixations and average duration of them per level: weakest performers.

**Participant ID**	**Age**	**Gender**	**Level 1**			**Level 2**			**Level 3**			**Level 4**			**Total**	

			**No. Fix.**	**Fix. Avg Duration (ms)**	**SD (ms)**	**No. Fix.**	**Fix. Avg Duration (ms)**	**SD (ms)**	**No. Fix.**	**Fix. Avg Duration (ms)**	**SD (ms)**	**No. Fix.**	**Fix. Avg Duration (ms)**	**SD (ms)**	**Time (s)**	**Correct Ans**
15	11	Female	125	169.91	80.86	180	167.95	74.16	209	168.85	71.15	245	167.82	69.23	171.33	9
16	9	Female	138	237.16	128.35	35	243.44	94.41	50	240.26	90.68	98	235.15	84.44	189.66	6
29	12	Male	91	174.38	76.58	38	185.29	91.79	17	179.01	70.67	36	176.08	70.79	192.56	9
36	8	Male	87	163.61	69.03	142	159.41	60.23	164	165.25	71.36	186	167.99	76.02	192.34	8

**Table 7:** Best vs. weakest performers by level: Mann–Whitney analysis of the results.

	**Level 1**		**Level 2**		**Level 3**		**Level 4**		**Global**	

	***p***	**Mann–Whitney**	***p***	**Mann–Whitney**	***p***	**Mann–Whitney**	***p***	**Mann–Whitney**	***p***	**Mann–Whitney**
		**U-Value**		**U-Value**		**U-Value**		**U-Value**		**U-Value**
Fix Number	0.33	6.0	0.05	2.0	0.23	5.0	0.03	1.0	0.03	1.0
Fix Avg. Time	0.23	5.0	0.33	3.0	0.15	4.0	0.09	3.0	0.01	0.0
Time	0.15	4.0	0.09	3.0	0.09	3.0	0.01	0.0	0.09	3.0
Correct Answers	0.42	8.0	0.43	8.0	0.01	0.0	0.01	5.0	0.09	3.0

**Table 8:** Performance comparison of feature selection algorithms using selected classifiers.

		**Feature Selection Algorithms**						

		**Select *k*-best**					**L1-Based**	**Hierarchical**
No. of Features	All	22	16	12	6	4	7	Btw10–14
Decision Tree	0.76 (±0.21)	0.80 (±0.18)	0.80 (±0.14)	0.81 (±0.15)	0.80 (±0.14)	0.80 (±0.15)	0.79 (±0.15)	0.82 (±0.17)
Random Forest	0.84 (±0.17)	0.87 (±0.11)	0.86 (±0.12)	0.87 (±0.11)	0.84 (±0.11)	0.83 (±0.14)	0.86 (±0.11)	0.88 (±0.11)
Extra Tree	0.80 (±0.18)	0.85 (±0.12)	0.82 (±0.14)	0.82 (±0.14)	0.81 (±0.14)	0.80 (±0.14)	0.83 (±0.14)	0.84 (±0.14)
AdaBoost	0.78 (±0.21)	0.85 (±0.14)	0.85 (±0.15)	0.84 (±0.15)	0.82 (±0.14)	0.81 (±0.15)	0.85 (±0.14)	0.86 (±0.13)

**Table 9:** Ensemble methods vs. decision trees: Mann–Whitney analysis of their accuracy.

**Ensemble Method**	**(accuracy)**	**Decision Tree (accuracy)**	***p***	**Mann–Whitney U-Value**
Random Forest	0.84		<0.001	2843.5
Extra Trees	0.8	0.76	0.003	3919.5
AdaBoost	0.78		0.02	4206

**Table 10:** All features vs. feature selection algorithms: Mann–Whitney analysis of their accuracy.

	**All Features *vs.* Selected K best (22 features)**		**All Features *vs.* L1-based (7 features)**		**All Features *vs.* Hierarchical (btw. 10–14 features)**	
		
	***p***	**Mann–Whitney**	***p***	**Mann–Whitney**	***p***	**Mann–Whitney**
		**U-Value**		**U-Value**		**U-Value**
Decision Trees	0.001	3550.5	0.002	3979	<0.001	3134.5
Random Forest	0.0007	3798.5	0.162	4201.5	0.003	3901.5
Extra Trees	<0.001	3228.5	0.0003	3638.5	<0.001	3308
AdaBoost	<0.001	3096.5	<0.001	3075	<0.001	2740.5
